# Gut Microbiota-Mediated Modulation of Cancer Progression and Therapy Efficacy

**DOI:** 10.3389/fcell.2021.626045

**Published:** 2021-09-08

**Authors:** Peng Cheng, Peiliang Shen, Yunlong Shan, Yu Yang, Rui Deng, Wenxing Chen, Yin Lu, Zhonghong Wei

**Affiliations:** ^1^Jiangsu Key Laboratory for Pharmacolgy and Safety Evaluation of Chinese Materia Medica, School of Pharmacy, Nanjing University of Chinese Medicine, Nanjing, China; ^2^Key Laboratory of Drug Metabolism and Pharmacokinetics, State Key Laboratory of Natural Medicines, China Pharmaceutical University, Nanjing, China

**Keywords:** gut microbiota, cancer, metabolism, gut immunity, immunotherapy, chemotherapy

## Abstract

The role of gut microbiota in the development of various tumors has been a rising topic of public interest, and in recent years, many studies have reported a close relationship between microbial groups and tumor development. Gut microbiota play a role in host metabolism, and the positive and negative alterations of these microbiota have an effect on tumor treatment. The microbiota directly promote, eliminate, and coordinate the efficacy of chemotherapy drugs and the toxicity of adjuvant drugs, and enhance the ability of patients to respond to tumors in adjuvant immunotherapy. In this review, we outline the significance of gut microbiota in tumor development, reveal its impacts on chemotherapy and immunotherapy, and discover various potential mechanisms whereby they influence tumor treatment. This review demonstrates the importance of intestinal microbiota-related research for clinical tumor treatment and provides additional strategy for clinical assistance in cancer treatment.

## Impact of Intestinal Microbiota on Tumorigenesis

**FIGURE 1:**
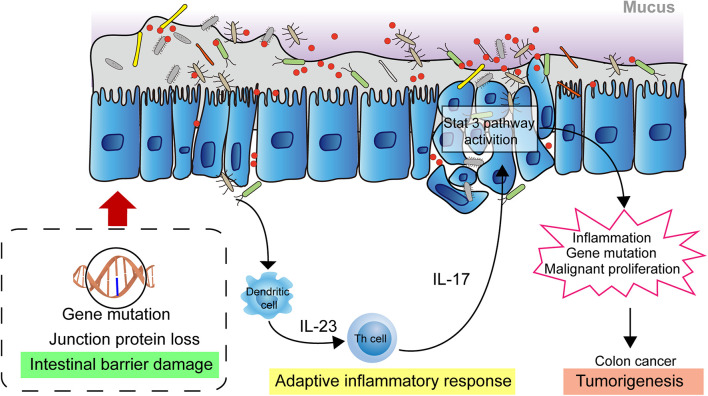
Intestinal microbial facilitating colon cancer progression. The damaged intestinal mucosal barrier permits the transposition of intestinal bacteria and metabolites from the lumen to the lamina propria which activates adaptive immune response. The releasing cytokines drives development of Th17 cells response. The response activates the STAT3, NF-κB signaling pathway in epithelial cells, promoting cell proliferation and invasion, leading to further destruction of mucosal integrity and increased inflammatory response. These biological events contribute to the development of colon cancer.

The homeostasis of the intestinal microbiota plays an important role in the normal physiological activities of the hosts, and microbiome imbalance greatly promotes tumorigenesis. In the past decade, substantial progress has been made in the investigation of the relationship between tumorigenesis and the role of microbiota. Investigations have shown that causes of cancer are associated with obesity, cardiovascular disease, type 2 diabetes, and carcinogenic chemicals, which are proven affected by the microbial community (Viennois et al., 2017; Cremonesi et al., 2018; Qin et al., 2018; Wu et al., 2019). Moreover, intestinal microbiota play a role in the pathogenesis of colorectal cancer. Studies have shown that microbiota indirectly affect the occurrence and development of colorectal cancer through inflammation and immune response (Honda and Littman, 2016; Chiaro et al., 2017). At present, the pathogenesis of colon cancer is mainly affected by epithelial gene mutation, mucosal integrity, intestinal microbiota, and inflammation. Gallimore and Godkin (2013) describe, in detail, the intestinal microorganism-mediated carcinogenic model and propose that the damage to the intestinal mucosal barrier integrity is key in the occurrence and development of colon cancer (Grivennikov et al., 2012; Gallimore and Godkin, 2013). Due to the destruction of the intestinal mucosal barrier, bacteria and their metabolites in the intestinal cavity are translocated to the lamina propria through the intercellular space of the epithelial cells, thus triggering an adaptive inflammatory response and the release of cytokines, such as IL-1, IL-6, and IL-23. This will activate downstream Th17 cells, promoting the release of IL-17 and further activating the inflammatory and proliferative pathways of epithelial cells, such as the STAT3 and NF-κB pathways, in turn, promoting cancer cell proliferation and invasion. This results in further destruction of mucosal integrity, aggravation of inflammatory reaction, and repeated inflammation of the colon, which directly factors in the occurrence and development of colon cancer (Figure 1) (Gallimore and Godkin, 2013; Chen et al., 2017; Dai et al., 2019).

The mucous layer is the first line of defense of the intestinal mucous barrier. Most of the microbial symbiosis in the human body occurs in the intestinal epithelial barrier, which greatly influences intestinal health. Researchers have found that microbes promote the normal structure and function of the intestine in aseptic mice, that is, the intestinal mucosa of aseptic mice is considerably thin, and intestinal epithelial cell proliferation is significantly reduced. Moreover, the production of mucin and other intestinal epithelial cell derivatives is also impaired. The thinner mucin layer negatively affects the protective function of the epithelial barrier, which allows contact between the host’s intestinal epithelium and exogenous substances, making it more vulnerable to foreign chemicals and pathogenic microbiota, thereby increasing the risk of colon cancer (Allaire et al., 2019).

The outer mucosal later is the habitat of symbiotic microbiota and the nutrient source of some microbiota. The microbial species inhabiting, and physiological functions of, the outer mucosal layer can affect the composition and structure of microbiota in the intestinal cavity. Nineteen strains from the phyla Bacteroidetes, Firmicutes, Actinomycetes, and Verrucobacteria have mucin-degrading ability (Tramontano et al., 2018); these strains mainly include *Akkermansia muciniphila* (Geerlings et al., 2018), *Barnesiella intestinihominis* (Desai et al., 2016), *Bacteroides thetaiotaomicron* (Xu et al., 2003; Comstock, 2009), *Bifidobacterium bifidum* (Turroni et al., 2011), *Bacteroides fragilis* (Hecht et al., 2017), *Bacteroides vulgatus* (Png et al., 2010), *Ruminococcus gnavus* (Owen et al., 2017), and *Ruminococcus torques* (Halmos et al., 2015). In addition to the physical mechanical barrier, the intestinal mucus layer also has an immuno-barrier function. Pathogenic microbiota are sensed by various types of pattern recognition receptors and antigen-presenting cells, and the colonic mucus is a repository of Immunoglobulin A (IgA). There are also some bactericidal proteins in the mucus that directly kill displaced microbiota (Xu et al., 2015; Honda and Littman, 2016; Chiaro et al., 2017; Allaire et al., 2019).

In addition to the direct effect on the intestinal tract, intestinal microbiota can indirectly affect tumor occurrence by influencing inflammation, immune function, and systemic metabolic function, in addition to their direct effect on the intestinal tract. Zhang Guodong’s team found that triclosan (dichlorophenoxy chlorophenol) could change the intestinal microorganism composition to reduce intestinal micromicrobiota diversity and the abundance of beneficial bacteria (*Bifidobacterium*, a known bacteria with anti-colitis), thereby promoting tumorigenesis through the development of colitis. Experimental results have shown that triclosan can disrupt the intestinal barrier function in the body, increase immune cell infiltration, cause the transfer of TLR4 ligands (such as lipopolysaccharides and other bacterial products) from the intestinal tract to the systemic circulation, and promote the activation of TLR4 signal, thus promoting the occurrence and development of colitis and colon cancer (Thaiss et al., 2016; Yang et al., 2018).

## Intestinal Microbiota and the Host’s Co-Metabolism Regulate Tumorigenesis and Tumor Progression

The intestinal microbiota composition grows and develops with the host and is affected by the complex interaction of the host genome, nutrition, and lifestyle. Intestinal microbiota is involved in the regulation of various host metabolic pathways, which affects the co-metabolism of host-micromicrobiota interaction (Nicholson et al., 2012). The host will rely on gut microbiota to increase the production of digestive and metabolic enzymes. Intestinal microbiota produce various metabolic components, which include fermentation products of undigested food and endogenous compounds produced by the host, to construct a natural metabolite microenvironment-intestinal epithelial mucus layer, so that microbial metabolites can enter and interact with the host cell to affect the host immune response and disease occurrence and development (Rooks and Garrett, 2016). The microbial community in the human body can prevent pathogen growth by producing beneficial microbial metabolites; however, the imbalance of metabolites in the body will result in side effects that induce the occurrence and development of cancer (Del Carmen Martínez-Jiménez et al., 2018). Further research on the co-metabolism of host and microbiota is required to optimize treatments that control the intestinal microbiota and is a potential treatment strategy in the prevention and cure of various diseases.

### The Impact of Intestinal Microbial Metabolites on Cancer Progression

The intestinal microbiota can use substances that cannot be digested by the small intestine, such as dietary fiber, and certain undigested sugars, proteins, and peptides. Consequently, the intestinal microbiota can also cooperate with the human body to form intestinal microbiota-host co-metabolites, such as short-chain fatty acids (SCFAs), vitamin H, and vitamin K (Lokody, 2014). These metabolites can be used as energy materials for intestinal microbes and can be transported to other parts of the body to stimulate cell growth, inhibit harmful microorganism growth, and participate in disease defense (Wu et al., 2016). When the composition, ratio, and quantity of the intestinal microbiota undergo pathological changes, it can lead to corresponding changes in the content and types of metabolites related to the microbiota, affecting the physiological functions of the host, and promoting the occurrence and development of tumors (Han et al., 2018). Therefore, changes in intestinal microbiota metabolites have important potential in tumor disease diagnosis.

#### Short-Chain Fatty Acids (SCFAs)

SCFAs are mainly produced by the microbial fermentation of undigested food, and its products are mainly acetate, propionate, and butyrate. Among the three major SCFAs, butyrate is the most important component that maintains colon health; it is primarily used as a direct energy source for colon cells. SCFAs plays a beneficial role in human intestinal health and affect colon health through various mechanisms. *In vivo* and *in vitro* studies have shown that SCFAs have anti-inflammatory and anticancer effects and plays an important role in maintaining the homeostasis of colon cell metabolism, protecting colon cells from external damage (McNabney and Henagan, 2017).

SCFAs can have a direct effect against cancer by inhibiting histone deacetylase (HDAC) and activating G protein-coupled receptors (GPCRs). HDAC inhibition by SCFAs is related to cell cycle arrest. Moreover, the microarray analysis of human colonic epithelial cells reveals that most butyrate-related genes are indeed involved. Butyrate can reduce the expression levels of the anti-apoptotic gene Bcl-2 and the pro-apoptotic protein Bax. Further, butyric and propionic acids can promote adenoma and cancer cell apoptosis by stimulating the expression of the cell cycle regulatory genes p53 and p21. Finally, butyric and propionic acids can promote cancer cell differentiation, inhibit colon cancer cell migration *in vitro*, and reduce the invasiveness of colon cancer cells. In addition, both activated GPR43 and GPR109a have anti-tumor effects by inhibiting proliferation and promoting the apoptosis of colon cancer cells, not related to HDAC inhibition (van der Beek et al., 2017). Furthermore, the high expression of SCFAs receptors in immune cells has indicated that SCFAs affect the expansion and production of Treg cells through the inhibition of SCFA-GPCR or HDAC, participating in intestinal immune regulation, and regulating and colon cancer development (El Kaoutari et al., 2013; Johansson et al., 2015).

Based on the above analysis, the role of SCFAs seems contradictory. It could provide energy for the growth and proliferation of normal cells, and inhibit cancer cell proliferation. However, current studies have shown that butyric acid generally could not reach the crypt cells, mainly providing energy for the colon cells in the anterior segment of the crypt. While the crypt structure is destroyed in the state of colon cancer, butyric acid could mediate the inhibitory effect of colon stem cells on colon cancer cells. SCFAs produced by intestinal microbiota play an active role in maintaining the normal state of the body, based on the cooperation between micromicrobiota and the body. Propionic and butyric acids may be involved in the above-mentioned tumor inhibition pathway (Alexander et al., 2017). Exploring the addition of probiotics and prebiotics in preventing or treating cancer can provide new ideas for its clinical treatment.

#### Amino Acids and Their Derivatives

In the distal human gut, proteins and peptides have three possible prospects: assimilated by the microbiota; as the substrate of microbial alienation metabolism, wherein its products enter the host portal circulation; or as intermediates of extensive microbial crosstalk and fecal excretion (Krautkramer et al., 2021). To a large extent, the degree of amino acid metabolism of intestinal microbiota depends on the utilization of substrate and cavity environments. It has been reported that the bacterial fermentation rate of protein (relative to carbohydrates) is greater in higher colonic pH and lower carbohydrate utilization (Krautkramer et al., 2021). SCFA production caused by the microbial protein degradation being significantly lower than that of carbohydrates. In addition, the decrease of organic acids leads to a higher pH value of the lumen, which in turn alters microbiota structure and function (Ratzke and Gore, 2018). In contrast, the low lumen pH, which is a result of the presence of SCFAs, is considered to inhibit bacterial proteases; eventually, fermentable carbohydrates drive bacterial growth, and bacterial protein assimilation then increases at the expense of fermentation (Stephen and Cummings, 1980; Birkett et al., 1996). To date, due to the complexity of intestinal microbial content, the complex interdependence between many hosts and these substrate metabolic pathways, and the technical limitations of metabolite source classification (host and microbiota), has limited research on intestinal microbial histone degradation to a certain extent (Sridharan et al., 2014). However, in recent decades, intestinal microbiota has recovered large amounts of energy from proteins and peptides that escape host digestion; this results in the synthesis of various bioactive compounds, some of which are potentially toxic, including SCFAs, bifunctional chelating agents, ammonia, phenol, indole, amines, sulfides, and N-nitroso compounds (Smith and Macfarlane, 1996).

Tryptophan is an essential amino acid that ismetabolized into indole derivatives, 5- hydroxytryptophan (5-HT) and kynurenines (kynurenine and its derivatives) by different pathways. Indolepropionic acid (IPA) inhibit the early development of breast cancer by acting on AHR and PXR (Sari et al., 2020), which is converted by *Bacteroides* spp., *Clostridium* spp., *Lactobacillus* spp., *Parabacteroides distasonis, Peptostreptococcus* spp. etc. (Dodd et al., 2017; Agus et al., 2018). Recent studies have shown that 5-HT enhances the activation of NLRP3 inflammasomes by acting on its ion channel receptor HTR3A and promotes tumor progression in colitis associated colorectal cancer mouse models (Li et al., 2021). The microbiota is also involved in regulating host 5-HT, and Some species grown in culture can produce 5-HT (Tsavkelova et al., 2006). Kynurenines are ligands for arylhydrocarbon receptor (AhR) ligands to promote cell migration and immune tolerance, thereby driving cancer progression (Cervenka et al., 2017). Indoleamine 2, 3-dioxygenase 1 (IDO1) is the rate-limiting step of tryptophan conversion to kynurenines. The gut microbiota, such as *Lactobacillus* spp., *Pseudomonas aeruginosa, Pseudomonas*, *Fluorescens* (Vujkovic-Cvijin et al., 2013; Agus et al., 2018), promotes the expression of IDO1, and IDO1 activity can also regulate the composition of the microbiome.

### Microorganism-Mediated Host Metabolism Affects Tumor Progression

Secondary bile acid is a primary bile acid catalyzed by intestinal microbiota, which is a substance that is converted by dehydroxyl group by the de-binding reaction. Secondary bile acids have a potential DNA-damaging ability, that is, a carcinogenic effect (Cao et al., 2017). In people with obesity, the dominant *Clostridium* members in the intestines can convert primary bile acids (such as chenodeoxycholic acid and cholic acid) into secondary bile acids (such as lithocholic acid and deoxycholic acid), which have a high affinity for bile acid receptors that affect multiple metabolism-associated processes. Hepatic sinusoidal endothelial cells expressed chemokine CXCL16 (CXCR6 ligand) to regulate the accumulation of NKT cells. Intestinal microbiota then enzymatically modify primary bile acidsto secondary bile acids, which could affect the process: primary bile acids increased the expression of CXCL16, while secondary bile acids had the opposite effect (Ma et al., 2018). Intestinal bacteria with this enzymatic reaction capacity have BSH enzymes, including *Lactobacillus* (Wang et al., 2012), *Bifidobacterium* (Kim et al., 2004), *Clostridium* spp. (Kim et al., 2004), *Listeria* (Begley et al., 2005), and *Enterococcus* (Wijaya et al., 2004). In addition, secondary bile acids can directly or indirectly affect the composition of intestinal microbiota. Current studies have shown that reduced abundance of *Clostridium labile* has an inhibitory effect on secondary bile acids, which can prevent liver tumors in mice (Winston and Theriot, 2016).

## Impact of Intestinal Microbiota on Tumor Immunotherapy and Chemotherapy

Spontaneous tumor remission in patients with severe bacterial infection has been reported for the past two centuries. Coley, a surgical oncologist from the United States, used *Streptococcus pyogenes* extract named Coley’s toxin to treat tumor patients at the late nineteenth century. Approximately 30% of lymphoma and sarcoma patients were cured, thus opening the door to tumor immunotherapy. In the history of tumor therapy development, chemotherapy agents emerged as the mainstay of present tumor therapy (Hoffman, 2012). Exploring the combination of intestinal microbiota with chemotherapeutic drugs and the interaction mechanism between them can provide better treatment innovations in clinical settings.

### Impact of Intestinal Microorganism-Mediated Chemotherapy Drugs on Tumor Treatment

Since the discovery of nitrogen mustard cytotoxicity in World War II, researchers have gradually developed cytotoxic chemotherapeutic agents, such as chemotherapeutic drugs. Nowadays, chemotherapeutic drugs remain as the main treatment of tumors in most clinics (Einhorn, 1985). Lehouritis et al. (2015) observed the potential effect of bacteria on the effectiveness of chemotherapeutic drugs against cancer cells *in vitro*, whereby the activities of 10 out of 30 tested drugs were found to be specifically inhibited by one or two bacteria, and the correlation analysis of HPLC and mass spectrometry revealed that bacterial contact leads to the biotransformation of drugs. Therefore, experimental results show a complex and dynamic interaction between chemotherapeutic drugs and microbiota.

Intestinal microbiota directly affect drug absorption and metabolism, and indirectly affect oral drug metabolism by regulating host gene expression. Compared with ordinary mice, it was found that the expression of certain members of the cytochrome P450 (Cyp450) gene family increased in aseptic mice livers. The expression of proteins from the Cyp2a, Cyp2b, and Cyp3a families, which are involved in heterogeneous steroid metabolism was increased, but that of other cytochromes was decreased. The fatty acid and arachidonic acid metabolism associated with members from the Cyp4a family involves heterogeneous biosensing receptors and transcription factors, such as androgen receptor, aryl hydrogen carbon receptor, and P450 oxidoreductase, which regulate target gene overexpression. Interestingly, the colonization of microbiota taken from routinely cultured ordinary mice in aseptic mice can restore the normal expression of related genes, and probiotic use in aseptic mice can also improve certain gene expression. These gene changes accelerate the metabolism of multiple drugs in aseptic mice (Jourová et al., 2017; Li et al., 2017). The role of microbiota in regulating drug metabolism and detoxification was also indirectly proven. Therefore, the heterogeneity of the therapeutic effect and toxicity of drugs on tumor patients can be exhibited from the differences in the composition and activity of intestinal microbiota among individual patients (Björkholm et al., 2009; Selwyn et al., 2015). In addition to oral drugs, several injected drugs are metabolized in the liver, and then excreted into the intestinal tract through bile, in which they are further metabolized and reabsorbed in the intestinal microbiota’ environment.

Additionally, intestinal microbiota are involved in the biochemical conversion of various drugs, including reduction, hydrolysis and functional group removal, such as N-oxide cleavage, proteolysis, denitrification, amine formation, hydrolysis, thiazole ring opening, and acetylation (Wilson and Nicholson, 2017). Microbiota also reduce drug absorption through physical binding and separation. At present, it has been shown that more than 40 types of exogenous chemicals (non-natural foreign chemicals) are metabolized by intestinal microbiota. However, among anticancer drugs, only misonidazole, a radiosensitizer, and irinotecan (also known as CPT-11), a topoisomerase I inhibitor for hydrolysis and depolymerization of methotrexate, are affected by intestinal microbiota (Haiser and Turnbaugh, 2013).

Most chemotherapeutic drugs have no specificity and generally produce significant toxicity for all cells and tissues exhibiting accelerated renewal (Sancho-Martínez et al., 2012). Platinum anti-tumor drugs, such as oxaliplatin and cisplatin, kill tumor cells by inhibiting DNA replication and targeting the cell membranes and mitochondria. In addition, they can cause severe enterotoxicity, nephrotoxicity, ototoxicity, and peripheral neuropathy. Chemotherapeutic drugs have a strong toxic effect, particularly on intestinal mucosal cells which show acute regeneration, and are damaging to intestinal barrier function, moreover, this damage causes microbiota and pathogens to enter the mesenteric lymph nodes and blood circulation, leading to septicemia and systemic inflammation (Hooper and Macpherson, 2010). Therefore, generating better methods to combine and operate biospecific molecules on the surface of the chemotherapeutic drugs in order to safely and effectively deliver the drugs to the tumor site is a limitation of the current research. The latest research uses genetic engineering to modify bacterial protoplasts and develop nano-vesicles without toxic outer membrane components, which can aid in the specific targeting of the tumor tissue by chemotherapeutic agents to improve drug safety and efficacy (Kim et al., 2017).

Briefly, intestinal microbiota play a critical role in drug metabolism, and the interaction between chemotherapeutic drugs and intestinal microbiota has a major effect. A significant reference for the development of chemotherapeutic drugs can be provided through a deeper understanding of the role and function of intestinal microbiota in the pathology and treatment of cancer.

### The Effect of Intestinal Microbiota on Tumor Immunotherapy

Tumor immunotherapy kills cancer cells and inhibits their proliferation through artificial intervention and mobilization of the body’s own immune system. It is the fourth tumor therapy after surgery, radiotherapy, and chemotherapy, with great potential for further development. Particularly, immune checkpoint inhibitors (ICIs), particularly CTLA-4 and PD-1 protein inhibitors, and adoptive cell therapy show good prospects in various tumor treatment.

Recently, ICIs have played an important role in tumor therapy. CTLA-4 inhibitor, the first immune checkpoint inhibitor, was discovered in 2011, with the approval of ipilimumab by the United States FDA. In 2014, nivolumab was approved as the first PD-1 inhibitor on the market worldwide. Within the next few years, a number of ICIs, including the antibody against PD-1 and PD-L1, were approved. The main indications include metastatic non-small cell lung cancer, melanoma, and urothelial carcinoma. At present, five PD-1 and PD-L1 antibodies have been approved by the US FDA, namely, nivolumab, pembrolizumab, atezolizumab, avelumab, and durvalumab (Clarke et al., 2018; Gong et al., 2018) (Table 1).

**TABLE 1:** List of ICIs.

Drugs	Mechanisms of action	Principal indication	Approval times	References
**Monoclonal antibody**				
1. Ipilimumab	CTLA-4	Unresectable or metastatic melanoma	2011	Billan et al., 2020
2. Nivolumab	PD-1	Melanoma and other indications	2014	Yau et al., 2020
3. Pembrolizumab	PD-1	Melanoma and lung cancer and other indications	2016	Pestana et al., 2020
4. Atezolizumab	PD-L1	Urothelial carcinoma	2016	Alhalabi et al., 2019
5. Avelumab	PD-L1	Metastatic merkel cell carcinoma and locally advanced or metastatic urothelial carcinoma	2017	Kim et al., 2020
6. Durvalumab	PD-L1	Locally advanced or metastatic urothelial carcinoma	2017	Kim et al., 2020
**Small-molecule drugs**				
7. AUNP-12	PD-1-PD-L1 interaction	Inhibition of Tumor growth and metastasis	–	Weinmann, 2016
8. Inhibitor of IDO	Kynurenine pathway	Inhibit immune evasion system of tumor cells	–	Mándi and Vécsei, 2012; Dounay et al., 2015; Gostner et al., 2015; Zak et al., 2015
9. Inhibitor of CD39 or CD73	Adenosine pathway	Enhances the effect of tumor vaccines during T cell activation	–	Bastid et al., 2013; Antonioli et al., 2014; Muller-Haegele et al., 2014; Young et al., 2014; Bhattarai et al., 2015
10. STING Activators	STING	Activates innate immunity and T cell recruitment factors	–	Dubensky et al., 2013; Fridlender et al., 2013; Gravekamp and Chandra, 2015; Wang et al., 2015
11. Toll-Like Activators	Toll-Like Receptors	Activates dendritic cells and natural killer cells	–	Hamm et al., 2009; Holldack, 2014; Mancini et al., 2014; Pradere et al., 2014
12. SyAM-Ps	Prostate-specific membrane antigen and Fc g receptor	Effectively recruits immune cells and acts as cytotoxic agents	–	McEnaney et al., 2014

With immunotherapy development, it has been found that small molecule drugs have greater advantages that might be complementary and potentially synergistic to large biological molecules in the immune system (Weinmann, 2016) (Table 1).

In addition, the role of microbiota is essential in immunotherapy. In 2015, the Gustave Roussy Cancer Center and Laurence found that gut microbiota composition determines the effectiveness of cancer immunotherapy represented by ICIs. In 2017, the two teams proved that microbiota *in vivo* play a decisive role in immunotherapy based on a large-scale analysis of patients with different types of cancer treated with PD-1 inhibitors (Sivan et al., 2015; Vétizou et al., 2015; Gopalakrishnan et al., 2018; Routy et al., 2018).

However, certain problems remain in tumor immunotherapy, such as the uncertainty of the curative effect, the narrow application scope, and immune system-related complications. Therefore, finding an optimal approach to immunotherapy is an important direction of clinical research. In 2015, Vétizou et al. discovered anticancer immunotherapy by CTLA-4 blockade that relies on the gut microbiota. Since then, the relationship between tumor immunotherapy and intestinal microbiota has become a popular research topic (Vesely and Schreiber, 2013; Bachireddy et al., 2015; Garrett, 2015; Segre, 2015; Topalian et al., 2015; Drewes et al., 2016; Tran et al., 2017).

#### Immune Checkpoint Inhibitor Responses May Be Affected by Gut Microbiota Composition

Clinical studies have confirmed that the progression-free survival and overall survival of patients with malignant tumors using antibiotics are significantly shorter than those of patients undergoing immunotherapy that did not use antibiotics, indicating the important role of the intestine in the tumor immunotherapy process (Derosa et al., 2018; Routy et al., 2018). Intestinal microbiota analysis revealed that the abundance of various types of *bifidobacteria* spp. increased significantly in mice with slow tumor growth, and anti-PD-1 therapy could have a significant effect. Useful microbiota in mice could be transferred to mice that lack them by fecal microbiota transplantation (FMT), or through feeding in the same nest. In addition, the anti-tumor effect of PD-L1 blockade was improved in mice that had an unfavorable intestinal microbiota through the oral administration of *Bifidobacterium*-containing probiotics. This effect predominantly benefits from the enhancement of DC maturation, which increases tumor-specific CD8^+^ T cell activity (Sivan et al., 2015). After anti-CTLA-4 treatment, the richness of intestinal micromicrobiota in mice changed significantly, mainly manifesting as a relative increase in *Bacteroidales* and *Burkholderiales* abundance, and a decrease in that of *Clostridiales*. Oral administration of *Bacteroides fragilis* with cyclophosphamide (*Bacteroides thetaiotaomicron*) or cephalosporin (*Burkholderia cepacia*) could trigger Th1 reaction and promote DC maturation, thus improving the efficacy of anti-CTLA-4 therapy. In germ-free and specific-pathogen-free mice treated with broad-spectrum antibiotics, the effect of anti-CTLA-4 therapy was significantly reduced, which could reverse the trend of the dominant microbiota of patients by FMT (Vétizou et al., 2015).

Researchers from the University of Texas MD Anderson Cancer Center have found that intestinal microbiota regulate the response of melanoma patients to PD-1 immunotherapy (Beaver et al., 2018). Through fecal microbial sample analysis and the study of different intestinal microorganism functions in the metagenome of patients, they found that there was a significant difference in intestinal micromicrobiota composition between melanoma patients who responded, and did not respond, to PD-1 immunotherapy and the immune response to tumors was significantly enhanced. To verify the conjecture, they transplanted the relevant beneficial bacteria and the feces of the respondent into the sterile mice with melanoma, and the same result was obtained.

**FIGURE 2:**
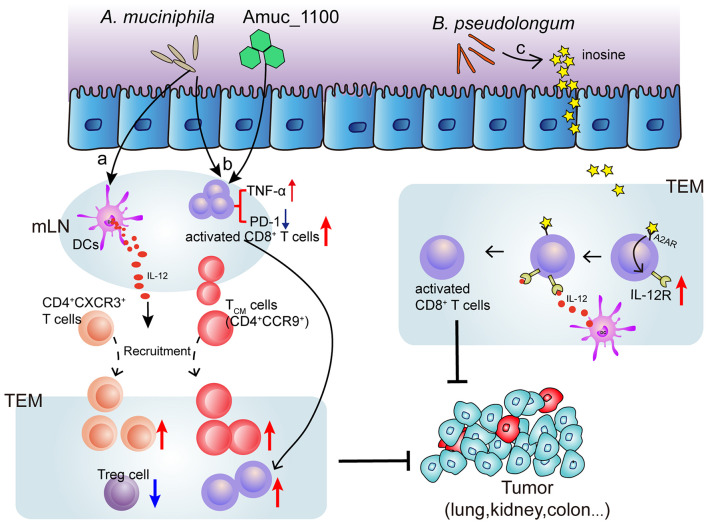
Mechanism of bacteria (*Akkermansia muciniphila* and *Bifidobacterium pseudolongum*) induced ICB efficacy enhancement. **(a)** Oral supplementation with *A. muciniphila* accelerates IL-12 secretion by dendritic cells (DCs) leading to restoring the recruitment of CXCR3^+^CD4^+^ and CCR9^+^ CD4^+^ T lymphocytes into tumor microenvironment (TEM), so that restoring the efficacy of PD-1 blockade. **(b)** *A. muciniphila* or a A purified membrane protein (Amuc_1100) from *A. muciniphila* treatment activates CD8^+^ T cells in mesenteric lymph nodes (mLN) and promote the recruitment of CD8^+^ T cells into TEM, representing by TNF-α upregulation and PD-1 downregulation. **(c)** Inosine, the metabolite from *B. pseudolongum*, co-stimulates A2A receptor (A2AR) leading to increased expression of IL-12 receptor on CD8^+^T cells, which further increases the T cell activation.

Several species of intestinal bacteria have been associated with enhanced efficacy of immune-checkpoint blockade (ICB). *Akkermansia muciniphil*a was correlated with increased immune cell infiltration in lung and kidney cancers, as CCR9^+^CXCR3^+^CD4^+^ T cells were recruited to the tumor bed in an interleukin-12-dependent manner and the ratio of CD4^+^ T cells to CD4^+^FoxP3^+^ T cells (Tregs) was increased (Routy et al., 2018). Oral administration of pasteurized *A. muciniphila* or its purified membrane protein Amuc_1100 reduced colon infiltration of macrophages and cytotoxic T lymphocytes (CTL) and improve colitis in mice. Moreover, they increased the number of CTL in colon and mesenteric lymph nodes, up-regulate the expression of TNF-α, inhibit the expression of PD-1, and increase the activation of CTL, which has an inhibitory effect on colitis associated colorectal cancer in mice (Wang et al., 2020). Furthermore, Mager et al. (2020) isolated three bacterial species, including *Bifidobacterium pseudolongum*, *Lactobacillus johnsonii*, and *Olsenella species*, that significantly enhanced the efficacy of immune checkpoint inhibitors (anti-CTLA-4 and anti-PD-1). Notably, intestinal *B. pseudolongum* modulated enhanced immunotherapy response through the production of the metabolite inosine (Mager et al., 2020) (Figure 2). However, the mechanisms whereby the microbiome enhances anti-tumor immunity are unclear.

#### Intestinal Microbiota Can Cause Immune Checkpoint Blockade-Associated Toxicity

Immune checkpoint inhibitors enhance the activity of anti-tumor T cells and are widely used in cancer therapy, but also cause immune-related adverse reactions, especially in the gastrointestinal tract (Soularue et al., 2018). The effect of intestinal microbiota on toxicity was studied in animal models and clinical cohorts. The melanoma patients treated with anti-CTLA-4 were rich in *Bacteroidetes* and various genetic pathways, involving polyamine transport and B vitamin synthesis, and no colitis occurred. This lack of toxicity could be related to the known effects of these bacteria. Interestingly, the bacteria related to reaction and toxicity have different taxa, some of which have overlapping characteristics. According to related studies, there was a higher risk of colitis in patients with a higher abundance of *Faecalibacterium prausnitzii* and a lower abundance of *Bacteroides* after anti-CTLA-4 treatment (Chaput et al., 2017). However, other studies have shown that a greater abundance of *Ruminococcaceae* family (including *Faecalibacterium* spp.) have been found in patients who responded to ICIs therapy (Gopalakrishnan et al., 2018). In contrast, bacterial taxa related to a poor response to ICIs are included in the order Bacteroidales of Bacteroidetes; however, a higher abundance of these classifications usually reduces the incidence of toxicity. In addition, a series of studies have shown that the intestinal microbiota can affect the therapeutic effects of immune checkpoints through a series of metabolites. Members of Firmicutes and *F. prausnitzii* produce SCFAs. These SCFAs enter the bloodstream through the damaged intestinal mucosa (Coutzac et al., 2020). It can reduce the effect of immune checkpoint suppression via the upregulation of CD80/CD86 on DCs and promoting the accumulation of tumor-specific T cells and memory T cells.

In summary, intestinal microbiota undeniably affect immunotherapy. Intestinal microbiota can regulate the anti-tumor immune response and the response to immune checkpoint inhibitors. These findings explain the therapeutic potential of intestinal microbiota in checkpoint blocking immunotherapy, which can greatly contribute to the treatment of tumors through further clinical and experimental studies (Gopalakrishnan et al., 2018).

#### The Role of Adoptive Cellular Immunotherapy Affected by the Intestinal Microbiota

Since the FDA first approved CAR-T therapy in 2017, adoptive cell therapy (ACT) has become a hotspot in current tumor immunotherapy research due to its remarkable efficacy in treating various tumors. Due to the recent approval of ACT, there are relatively few clinical studies on its effects on the intestinal microbiota. In 2007, Paulos et al. (2007) first demonstrated that in mouse tumor models, intestinal microbiota can be induced to translocate to the mesenteric lymph nodes under the action of systemic radiotherapy; then, the efficacy of ACT was enhanced through TLR4 signal transduction. After antibiotic treatment in mice, the efficacy of ACT was greatly reduced. After supplementation with bacterial lipopolysaccharide, the anti-tumor response of ACT was enhanced (Paulos et al., 2007). This phenomenon was found in clinical studies of metastatic melanoma patients, and the efficacy of ACT was better in patients who received radiotherapy pretreatment. Further, Uribe-Herranz et al. (2018) reported that in the process of ACT for treating mouse tumors, the treatment efficacy in vancomycin-treated mice was significantly better than that of untreated mice. However, the effect of ACT after treatment with neomycin and metronidazole was lower than that of untreated mice. Compared with the treated group, there was no significant change; 16S rRNA sequencing of mouse feces found that several genes from the members of Bacteroidetes were significantly different following vancomycin treatment, including bacteria from *Bacteroides* and *Parabacteroides*. Further research found that the mechanism underlying the action of ACT may involve the increased abundance of systemic CD8α + DCs, which further promotes IL-12 expression and enhances the efficacy of ACT. In summary, these results revealed a certain correlation between the intestinal microbiota and the therapeutic effects of ACT; however, the specific microbiota that exert these effects remain unknown. With the continuous expansion of ACT-related research and the wide range of clinical applications, more in-depth research will reveal potential gut microbiota-related targets for improving the therapeutic effects of ACT.

## Summary and Outlook

With the in-depth study of intestinal microbiota, the correlation between intestinal microbiota and tumorigenesis will be revealed. The intestinal microbiota plays an important role in tumorigenesis and tumor progression, and simultaneously affects the effectiveness of chemotherapy. In recent years, increasing attention has been paid to the relationship between intestinal microbiota and tumor-related health problems, which mainly benefits from the rapid development of microtechnology, including the second and third generations of high-throughput sequencing, such as 16S rRNA sequencing and PCR-DGGE, that can determine the DNA of microbial specimens. Real-time fluorescence quantitative PCR was used to quantitatively study the intestinal microbiota and explore the microbial diversity. The bacteria in intestinal microbiota were obtained by anaerobic culture, and intestinal microorganism function was studied at the strain level. Fecal transplantation technology verifies the mechanism of intestinal microbiota through the forward and reverse experiment.

In current studies, the effects of intestinal microbiota on tumors and immunity have been observed; however, the mechanism underlying these effects remain unclear. In addition, there are still challenges associated with studying how intestinal microbiota regulation improves the effects of tumor immunotherapy. At present, the components of intestinal microbiota that are most conducive to promoting anti-tumor immune response remain unclear. Furthermore, there are various treatments to modify the intestinal microbiota, which need to be carefully tested in clinical trials. After fully understanding these interactions, optimization of intestinal microbiota regulation for enhancing the host’s anti-tumor immunity and for potentially improving immune surveillance can be achieved.

At present, only a few types of bacteria have been reported to be related to cancer, and great potential exists to explore the relationship between microbiota and tumorigenesis and tumor progression. The technologies to study these aspects are improving with the emergence of new treatments, such as the above-mentioned fecal transplantation, i.e., transplanting feces from healthy people to patients, which can have a good therapeutic effect and broaden the ideas of clinical treatment.

In future research, with improvements in microtechnology and analytical technology, we believe that more cancer-related target microbiota will be discovered, providing new methods and ideas for the clinical treatment of malignancies.

## Author Contributions

ZW and YL: conceptualization and design the review, review final version approval, and funding acquisition. PC, RD, YS, and WC: bibliographic research. PC, PS, and YY: writing—original draft preparation. PS, PC, YS, and ZW: the table and figure design. WC and YL: supervision. All authors contributed to the article and approved the submitted version.

## Conflict of Interest

The authors declare that the research was conducted in the absence of any commercial or financial relationships that could be construed as a potential conflict of interest.

## Publisher’s Note

All claims expressed in this article are solely those of the authors and do not necessarily represent those of their affiliated organizations, or those of the publisher, the editors and the reviewers. Any product that may be evaluated in this article, or claim that may be made by its manufacturer, is not guaranteed or endorsed by the publisher.
